# A novel use for Levey-Jennings charts in prenatal molecular diagnosis

**DOI:** 10.1186/s12920-020-00758-1

**Published:** 2020-07-31

**Authors:** Binghuan Weng, Ya-li Xu, Jun Ying, Hao-kun Yang, Lan Su, Yan-mei Yang, Min Chen

**Affiliations:** 1 The Key Laboratory of Reproductive Genetics, Department of Obstetrics and Gynecology, Women’s Hospital, School of Medicine, Zhejiang University, 1 Xueshi Road, Hangzhou, 310006 Zhejiang China; 2 Department of Clinical Laboratory Research, Shulan Hospital, Zhejiang University, 848 Dongxin Road, Hangzhou, 310006 Zhejiang China

**Keywords:** Aneuploidy, Molecular diagnosis, Prenatal diagnosis, Fluorescence in situ hybridization, Quality control, Standardization

## Abstract

**Background:**

The goal of this study was to determine whether Levey-Jennings charts, which are widely used in clinical laboratories, can be used to create standardized internal quality controls (IQCs) for prenatal molecular diagnosis.

**Methods:**

Aneuploid amniocyte lines with trisomy 13, 21, and 18, and 47,XXY were established by transfection with SV40LTag-pcDNA3.1(−)and combined at different ratios to generate aneuploidy chimeric quality-control cell mixtures A to H. These quality-control cells were then used to calculate the $\overline{\mathrm{X}}$, $\overline{\mathrm{X}}$ ±1 standard deviation (SD), $\overline{\mathrm{X}}$ ±2 SD, and $\overline{\mathrm{X}}$ ±3 SD values to develop standardized IQCs for methods used for the prenatal diagnosis of aneuploidies such as FISH.

**Results:**

Methods for constructing aneuploid amniocyte lines were developed and a set of quality-control cells (A-H) were prepared. The $\overline{\mathrm{X}}$ ±1 SD, $\overline{\mathrm{X}}$ ±2 SD, and $\overline{\mathrm{X}}$ ±3 SD values of these quality-control cells for trisomy 13 and 21 were 10.2 ± 1.7, 10.2 ± 3.4, and 10.2 ± 5.1, and 90.3 ± 2.3, 90.3 ± 4.6, and 90.3 ± 6.9, respectively. Based on the values and Levey-Jennings charts, a set of standardized IQCs for prenatal diagnosis such as FISH were established.

**Conclusions:**

This method resolves the problems of a shortage of quality-control materials and a lack of quality-control charts in prenatal molecular diagnosis such as NIPT, NGS, aCGH/SNP, PCR, and FISH. Levey-Jennings chart-based IQCs for prenatal diagnosis such as FISH can be used to easily monitor whether IQC results are within acceptable limits, and then infer whether the diagnostic results for clinical samples are reliable. We expect that this standardized IQC will be useful for a wide range of molecular diagnostic laboratories.

## Background

With the widespread development of molecular diagnostic technologies such as aneuploidy detection by fluorescence in situ hybridization (FISH) [1], noninvasive prenatal testing (NIPT) [2], and QF-PCR technique [3], standardized quality control is urgently needed to ensure that prenatal molecular diagnoses are accurate and reliable [3–5]. In general, the most important properties for quality control materials are as follows: they must behave like the real samples, and they must be available in sufficient quantity for developing internal quality control (IQC) charts to be used for monitoring quality in real time. At present, standardized IQCs are not easily available for prenatal molecular diagnosis because Levey-Jennings charts have not been used in molecular biology laboratories, and there is an insufficient supply of characterized paired materials such as cells with trisomy 18 and 13 in the cell line repository [6–8].

In 1931, Shewhart proposed the use of control charts for monitoring quality in manufacturing operations [9–13]. He defined values greater than 3S from the mean as indicating significant variation from standard quality. Over the past few decades, control charts have come to be widely used for IQC in clinical laboratories [10–14]. Levey and Jennings recommended that Shewhart’s control charts be used in clinical laboratories to provide a constant means of checking the reliability of the numerous tests run each day, and to make it possible to determine at a glance whether errors of analysis are beyond the permitted statistical variation defined by the procedure created in 1950 [10]. The control limit for Levey-Jennings charts is, as it was for Shewhart, 3S. Henry described the use of 1S, 2S, or 3S control limits and 95% confidence limits in 1959 [13], while Westgard et al. provided definitive guidelines for the interpretation of a control result that is 1S, 2S, or 3S from the mean in 1977 [14]. However, to date there have been few reports on the application of Levey-Jennings charts to prenatal molecular genetic diagnosis.

In the current study, we developed a set of aneuploidy chimeric quality-control cells (A-H) by immortalizing aneuploid amniocytes via SV40LTtransfection to address the lack of quality-control materials for methods used in prenatal diagnosis such as NIPT, NGS, and FISH [6–8]. More importantly, we developed a Levey-Jennings chart-based IQC system for detecting fatal aneuploidies by FISH by testing these quality-control cells(A-H) and clinical samples under the same experimental conditions. This novel use of control charts for monitoring prenatal diagnostic quality resolves the problem of the lack of adequate Levey-Jennings chart-based IQCs in molecular genetic laboratories, and should be widely applicable.

## Methods

### Establishment of immortalized aneuploid amniotic fluid cell lines

The SV40LTag-pcDNA3.1(−) recombinant vector was constructed and transfected into PT67 cells (ATCC CL-12284) using liposome transfection reagent to immortalize the cells [15]. The SV40LT vector produced from the immortalized PT67 cells was used to transfect primary amniocytes exhibiting trisomy 13, 18, trisomy 21, or 47,XXY to establish their immortalized amniotic fluid cell lines. After selection using G418, the immortalized amniotic fluid cell lines with passage numbers between 10 and 15 were harvested and preserved in liquid nitrogen at − 196 °C. These immortalized human amniotic fluid cell lines including trisomy 13, 18, trisomy 21, or 47,XXY were used in our study. All experiments were performed in accordance withthe Declaration of Helsinki and approved by the Insititutional Review Board of Zhejiang University School of Medicine Women’s Hospital, China (approve no. 20160030, 8 April 2016).

### Preparation of quality-control cells (A-H)

The trisomy 13, 18, and 21, and 47,XXY amniotic fluid cell lines were suspended at a density of 1 × 10^5^/ml. The trisomy 13 and 21 cell suspensions were mixed at ratios of 1:9, 3:7, 7:3, and 9:1 to prepare the quality-control cells A, B, C, and D containing 10, 30, 70, and 90% trisomy 13 and 90, 70, 30, and 10% trisomy 21 cells, respectively. Similarly, the trisomy 18 and 47,XXY cell suspensions were mixed to prepare the quality-control cells E, F, G, and H containing 10, 30, 70, and 90% trisomy 18 and 90, 70, 30, and 10% 47,XXY cells, respectively.

### Analysis of quality-control cells for application to FISH

A FISH kit was purchased from Jinpujia Company (Beijing, China). In this kit, GLP13 (green) is located at 13q14 and GLP21 (red) is located at 21q22. The 18/X/Y trichrome probes localize to the centromeres of chromosomes 18, X, and Y and show sky blue, green, and red fluorescence, respectively. The cells were fixed in a mixture of methanol and acetic acid (3:1) and then stained according to the manufacturer’s instructions. Technicians from the prenatal diagnosis laboratory of the Women’s Hospital, School of Medicine, Zhejiang University, and the Department of Clinical Laboratory Research, Shulan Hospital, Zhejiang University, counted 100 cells with obvious target hybridization (fluorescence) signals under a fluorescence microscope for each group of clinical samples (primary amniocytes) and quality-control cells. The quality-control cells (A-D) ratios for trisomy 13 or 21 hybridization signals were determined each day for 20 consecutive days to generate 20 datasets. Similarly, the quality-control cells (E-H) ratios for trisomy 18 or 47,XXY hybridization signals were determined. The $\overline{\mathrm{X}\ }$ ±1 SD, $\overline{\mathrm{X}\ }$ ±2 SD, and $\overline{\mathrm{X}\ }$ ±3 SD values for the datasets for each group of quality-control cells were calculated according to the formulas $\overline{\mathrm{X}\ }$ = $\frac{\sum \mathrm{X}}{\mathrm{n}}$ and S= $\sqrt{\frac{\sum {\left(\mathrm{X}-\overline{\mathrm{X}}\right)}^2}{\mathrm{n}-1}}$ to establish Levey-Jennings chart-based IQCs for aneuploidy detection.

### Plotting and applying Levey-Jennings chart-based IQCs

As an example, the Levey-Jennings charts *x*-axis was numbered 1–31 to represent 31 days, and the *y*-axis was labeled $\overline{\mathrm{X}\ }$, $\overline{\mathrm{X}\ }$ ±1 SD, $\overline{\mathrm{X}\ }$ ±2 SD, and $\overline{\mathrm{X}\ }$ ±3 SD. Based on the ratio of trisomy 13 to trisomy 21 cells in quality-control cells (A) detected by FISH and on the Levey-Jennings charts [9–13], the $\overline{\mathrm{X}\ }$, $\overline{\mathrm{X}\ }$ ±1 SD, $\overline{\mathrm{X}\ }$ ±2 SD, and $\overline{\mathrm{X}\ }$ ±3 SD values for trisomy 13 were plotted on the Levey-Jennings chart *y*-axis to generate the Levey-Jennings chart-based IQC for trisomy 13 (Fig. 2a). The Levey-Jennings chart-based IQC for trisomy 21 was created in the same way (Fig. 2b). For clinical application, the trisomy 13 and 21 ratio in quality-control cells (A) and in clinical samples were detected by FISH under the same experimental conditions, and the detection results for trisomy 13 in quality-control cells (A) were plotted on the Levey-Jennings chart-based IQC (Fig. 2a) to generate the experimental application of Levey-Jennings chart-based IQC for trisomy 13 detected by FISH (Fig. 2c). The experimental application of Levey-Jennings chart-based IQC for trisomy 21 detected by FISH was created in the same way (Fig. 2d). Thus, we can monitor whether the detection results for trisomy 13 and 21 in quality-control cells (A) exceeded $\overline{\mathrm{X}\ }$ ±2 SD or $\overline{\mathrm{X}\ }$ ±3 SD (and thus the IQC limit for the clinical reference controls), and then infer whether the test results for the clinical samples processed that day were reliable. Similarly, quality-control cells(B–H) were generated to monitor systematic or accidental laboratory errors.

### Interpretation of control results

Clinical reference controls are considered to be outside of acceptable limits in the following circumstances [13, 14]: a, if one control observation is more than 3S beyond either side of the mean (1–3 s); b, if two consecutive control observations are more than 2S from the mean and both observations are on the same side of the mean (2–2 s); c, if four consecutive control observations exceed 1S and all are on the same side of the mean (4–1 s); d, if the control values lie on opposite sides of the mean and the difference between the largest and the smallest observations exceeds 4S (R-4 s); e, if 10 consecutive control observations fall on the same side of the mean (10X); f, if one control value exceeds 2S on either side of the mean, which is not considered outside of acceptable limits, but a “warning sign” (1–2 s).

## Results

### Establishment of amniocyte lines

**Fig. 1:**
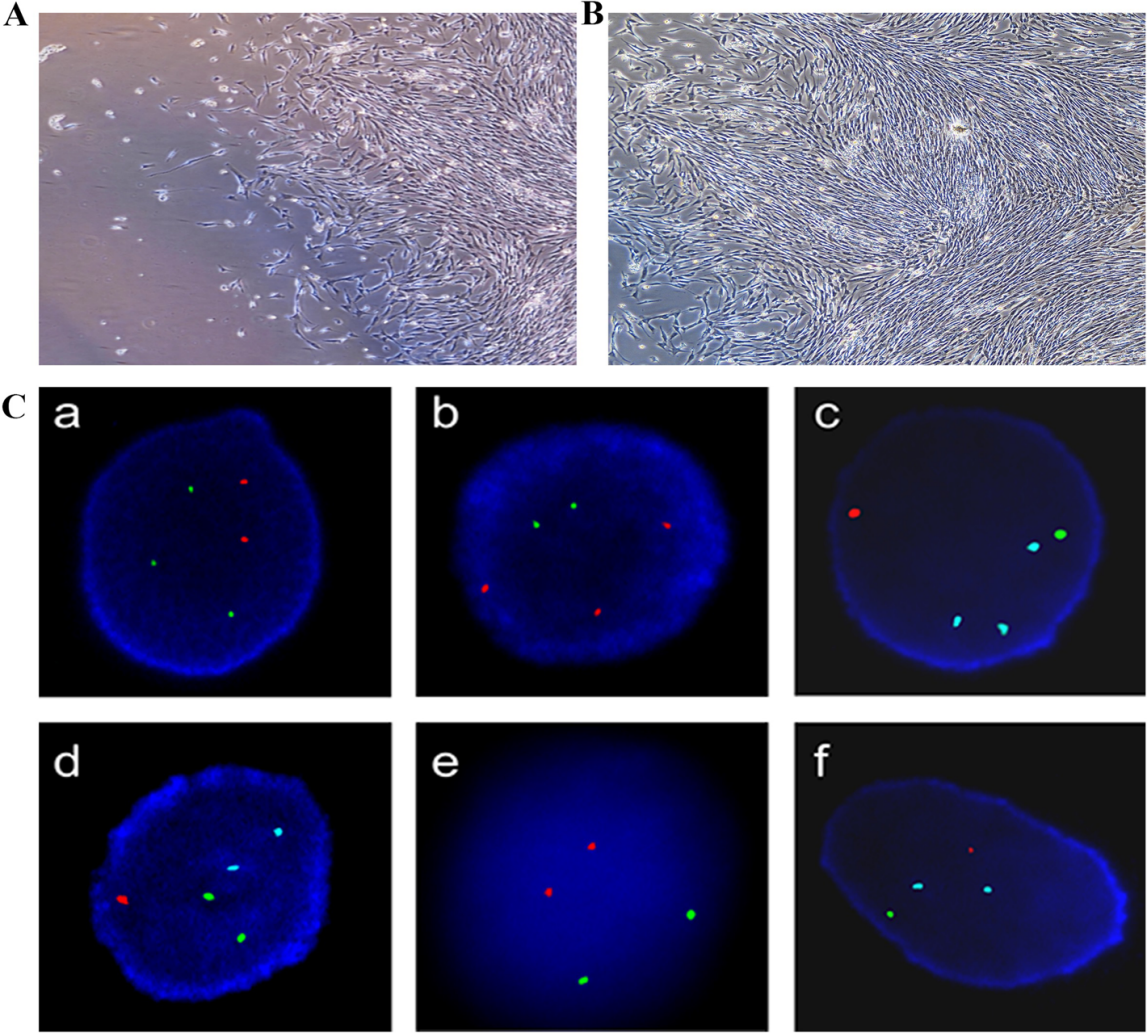
Established amniocyte lines and their hybridization signals. **a** Primary amniocytes were transfected with SV40LT and positive clones were obtained after selection with G418 (inverted microscope; × 40). **b** Monolayer adherent spindle-shaped amniocyte lines were formed when cultured in DMEM containing 10% fetal bovine serum (inverted microscope; × 40). **c** Comparison of quality-control cells and primary amniocytes. **a** and **b** show hybridization signals of trisomy 13 and 21 in quality-control cells, respectively. **c** and **d** show the signals of trisomy 18 and XXY in quality-control cells, respectively. The number of sky blue, green, and red signals represent the number of chromosomes 18, X, and Y, respectively. **e** and **f** show the signals of the 13/21 two-color probe and 18/X/Y trichrome probe in primary cells, respectively

Primary amniocytes with trisomy 13, 21, and 18, or 47,XXY were transfected with SV40LTag-pcDNA3.1(−)and then selected for in G418-containing medium (50–400 mg/L) for 7–15 days to obtain positive cell clones (Fig. 1A). When passaged in DMEM containing 10% fetal bovine serum, these cells grew as spindle-shaped adherent cells (Fig. 1B).

### Components of quality-control cells (A-H)

Quality-control cells(A–H) were prepared from amniocyte lines that had been immortalized, passaged, and frozen at − 196 °C in liquid nitrogen. In total, there are eight quality control samples: A, B, C, D, E, F, G, and H (Table 1). quality-control cells (A-D) are used for quality control of tests for trisomy 13 and 21. Theoretically, these samples contain 10, 30, 70, and 90% trisomy 13 cells and 90, 70, 30, and 10% trisomy 21 cells, respectively (Table 1). quality-control cells (E-H) are used for trisomy 18 and XXY, which are at the same ratios as the quality-control cells (A-D) (Table 1).

**Table 1:** Analysis of aneuploidies in quality control cells to establish internal quality controls (IQCs) for molecular genetic diagnosis ($\overline{\mathrm{X}\ }$ /SD/ $\overline{\mathrm{X}\ }$ ±1SD/2SD/3SD)

Group	Aneuploidy	Test (time)	Theoretical Ratio (%)	$\overline{\mathbf{X}\ }$(***n*** = 20)	SD	$\overline{\mathbf{X}\ }$±1SD	$\overline{\mathbf{X}}$±2SD	$\overline{\mathbf{X}}$±3SD
A	trisomy 13	20	10	10.2	1.7	10.2 ± 1.7	10.2 ± 3.4	10.2 ± 5.1
	trisomy 21	20	90	90.3	2.3	90.3 ± 2.3	90.3 ± 4.6	90.3 ± 6.9
B	trisomy 13	20	30	30.4	2.4	30.4 ± 2.4	30.4 ± 4.8	30.4 ± 7.2
	trisomy 21	20	70	69.8	2.6	69.8 ± 2.6	69.8 ± 5.2	69.8 ± 7.8
C	trisomy 13	20	70	70.3	2.4	70.3 ± 2.4	70.3 ± 4.8	70.3 ± 7.2
	trisomy 21	20	30	30.8	2.1	30.8 ± 2.1	30.8 ± 4.2	30.8 ± 6.3
D	trisomy 13	20	90	90.1	2.4	90.1 ± 2.4	90.1 ± 4.8	90.1 ± 7.2
	trisomy 21	20	10	10.1	1.4	10.1 ± 1.4	10.1 ± 2.8	10.1 ± 4.2
E	trisomy 18	20	10	10.3	1.9	10.3 ± 1.9	10.3 ± 3.8	10.3 ± 5.7
	47,XXY	20	90	90.6	2.4	90.6 ± 2.4	90.6 ± 4.8	90.6 ± 7.2
F	trisomy 18	20	30	30.0	2.6	30.0 ± 2.6	30.0 ± 5.2	30.0 ± 7.8
	47,XXY	20	70	69.8	2.5	69.8 ± 2.5	69.8 ± 5.0	69.8 ± 7.5
G	trisomy 18	20	70	70.1	2.4	70.1 ± 2.4	70.1 ± 4.8	70.1 ± 7.2
	47,XXY	20	30	29.9	2.6	29.9 ± 2.6	29.9 ± 5.2	29.9 ± 7.8
H	trisomy 18	20	90	90.6	2.6	90.6 ± 2.6	90.6 ± 5.2	90.6 ± 7.8
	47,XXY	20	10	10.1	1.3	10.1 ± 1.3	10.1 ± 2.6	10.1 ± 3.9

### Validation of quality-control cells fluorescence signals

Chromosomes 13, 21, 18, X, and Y in quality-control cells (A–H) were validated using a 13/21 two-color probe and an 18/X/Y trichrome probe. These probes both yielded positive fluorescence signals (Fig. 1a–d) that were consistent with the fluorescence signals for the primary amniocytes (Fig. 1e–f).

### Acquisition of IQC datasets

We prepared quality-control cells (A–D) for detection with the 13/21 probe and quality-control cells (E–H) for detection with the 18/X/Y probe. Quality-control cells A, B, C, and D contained 10, 30, 70, and 90% trisomy 13 cells and 90, 70, 30, and 10% trisomy 21 cells, respectively, while quality-control cells E, F, G, and H were composed of 10, 30, 70, and 90% trisomy 18 cells and 90, 70, 30, and 10% 47,XXY cells, respectively. Quality-control cells (A–H) and the test samples were analyzed 20 times under the same conditions to obtain datasets ($\overline{\mathrm{X}\ }$ /SD) for IQC (Table 1). Based on these datasets, the $\overline{\mathrm{X}\ }$ ±1 SD, $\overline{\mathrm{X}\ }$ ±2 SD, and $\overline{\mathrm{X}\ }$ ±3 SD values were calculated for trisomy 13 and 21 and trisomy 18 and 47,XXY (Table 1) to establish Levey-Jennings chart-based IQC.

### Establishing the Levey-Jennings chart-based IQC

**Fig. 2:**
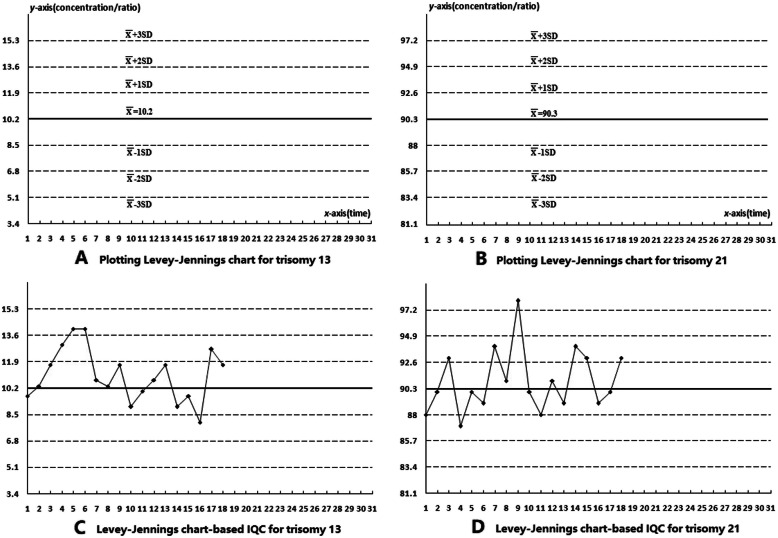
Levey-Jennings chart-based IQC for aneuploid detection. In Levey-Jennings charts (Fig. 2**a**) and (Fig.2**b**), quality-control data are plotted in the form of a graph in which time (dates) is represented on the *x*-axis and the numerical value (concentration/ratio) of the quality-control test is represented on the *y*-axis. The charts contain several reference lines including one reference line for the mean ($\overline{\mathrm{X}\ }$) and three reference lines on either side of the mean, representing standard deviation limits (1 SD, 2 SD, and 3SD). For quality-control cells (**a**), the values of $\overline{\mathrm{X}\ }$, $\overline{\mathrm{X}\ }$ ±1 SD, $\overline{\mathrm{X}\ }$ ±2 SD, and $\overline{\mathrm{X}\ }$ ±3 SD of trisomy 13 ($\overline{\mathrm{X}\ }$ =10.2, SD = 1.7) and 21 ($\overline{\mathrm{X}\ }$ =90.3, SD = 2.3) analyzed 20 times were plotted on the *y*-axis in Levey-Jennings charts (Fig. 2**a**) and (Fig. 2**b**), respectively. Then, the quality-control cells (**a**) and clinical samples were analyzed under the same experimental conditions and the results of each analysis of trisomy 13 were plotted in Levey-Jennings chart (Fig. 2**a**) to construct Levey-Jennings chart-based IQC for trisomy 13 (Fig. 2**c**, the values for the 5th and 6th exceeded $\overline{\mathrm{X}\ }$ + 2 SD), and the results of trisomy 21 were plotted in Levey-Jennings chart (Fig. 2**b**) to construct Levey-Jennings chart-based IQC for trisomy 21 (Fig. 2**d**, the values for 9th exceeded $\overline{\mathrm{X}\ }$ ±3 SD)

For quality-control cell (A), the $\overline{\mathrm{X}\ }$, $\overline{\mathrm{X}\ }$ ±1 SD, $\overline{\mathrm{X}\ }$ ±2 SD, and $\overline{\mathrm{X}\ }$ ±3 SD values for trisomy 13 and 21 over 20 days of detection were used to draw Levey-Jennings charts to establish the corresponding Levey-Jennings chart-based IQC for trisomy 13 (Fig. 2a) and Levey-Jennings chart-based IQC for trisomy 21 (Fig. 2b). Similarly, Levey-Jennings chart-based IQC were established for trisomy 13, 21, and 18, and 47,XXY in quality-control cells (B–H).

### Applying Levey-Jennings chart-based IQC for aneuploid detection

Quality-control cell (A) and clinical samples were analyzed for 18 days under the same conditions (Table 2). The results from each trisomy 13 and 21 test for quality-control cell (A) were plotted on Levey-Jennings chart for trisomy 13 (Fig. 2a) and for trisomy 21 (Fig. 2b) to apply the Levey-Jennings chart-based IQC for trisomy 13 (Fig. 2c) and for trisomy 21 (Fig. 2d), respectively. The results from the day 5 and day 6 tests of trisomy 13 for quality-control cell (A) were 14%, which exceeded $\overline{\mathrm{X}\ }$ ±2 SD (13.6%), indicating unreliable results from the clinical samples (Table 2 and Fig. 2c) [10–12]. Moreover, the day 9 test result for trisomy 21 in quality-control cell (A) was 98%, exceeding $\overline{\mathrm{X}\ }$ ±3 SD (97.2%), which also indicated unreliable results for the clinical samples (Table 2 and Fig. 2d) [10–12]. Similarly, the results from quality-control cells (B–H) corresponded with the Levey-Jennings chart-based IQC for aneuploidy detection by FISH.

**Table 2:** Aneuploidies in quality control cells (A and F) determined by applying molecular genetic diagnosis IQCs based on Levey-Jennings charts

Aneuploidy	Probe	Quality control cells (A and F)–based IQC results over 18 consecutive days																	
		1	2	3	4	5	6	7	8	9	10	11	12	13	14	15	16	17	18
Trisomy 13	GLP13/21	10	10	12	13	14	14	11	10	12	9	10	11	12	9	10	8	13	12
Trisomy 21	GLP13/21	88	90	93	87	90	89	94	91	98	90	88	91	89	94	93	89	90	93
Trisomy 18	18/X/Y trichrome	31	34	28	27	30	27	38	29	30	34	22	29	33	28	27	33	28	32
47,XXY	18/X/Y trichrome	68	69	70	69	61	68	67	70	68	71	69	73	68	70	68	76	69	73

## Discussion

Aneuploidy is one of the most common birth defects, and is mainly detected by prenatal molecular diagnosis [1–3]. The importance of quality control in molecular diagnosis has gained considerable attention in local and international studies in recent years [3–5, 16, 17]. For example, Hardwick *etal* [4]. indicated that the informed use of reference standards can ensure the rigorous analysis of next generation sequencing (NGS) and is essential for its future clinical use. Stromcm et al. [5] proposed improving the accuracy of prenatal screening with DNA copy-number analysis. However, quality control materials that have properties identical to clinical samples and that are applicable to a wide range of methodologies are still not available for aneuploidy screening, because there is an insufficient supply of characterized paired samples for trisomy 18 and 13 in the cell line repository (the Coriell Institute for Medical Research) [8].

To overcome the shortage of quality control materials [6–8], we developed a set of suitable quality-control cells that comprise the common aneuploidies trisomy 13, 21, and 18, and 47,XXY based on immortalization of amniocytes by SV40LT-transfection [15] (Fig. 1A-B). The established aneuploid amniocyte lines can be amplified in vitro for more than 350 generations [15, 18], while still preserving the same differentiated phenotype and biological characteristics [19]. As shown in Fig. 1C, the amniotic cell lines selected for using G418 and passaged 10–15 times were verified using a 13/21 two-color probe and an 18/X/Y trichrome probe, and showed clear positive signals for chromosomes 13, 21, 18, X, and Y, which were consistent with the fluorescence signals of the primary cells. These results validated the genomic stability of the aneuploid amniotic cell lines transfected with SV40LT [19]. These cells lines can be used as quality control materials for aneuploidy detection by FISH and to generate highly biomimetic control materials for NIPT and single-gene disorders based on enzymatic digestion for use in test validation, IQC, and proficiency testing in clinical or laboratory settings [8].

Our main purpose was to create a novel use for Levey-Jennings charts in molecular biology laboratories by developing characterized paired quality-control cells. Levey-Jennings charts, which were initially used by Westgard to monitor the quality of manufacturing operations, were recommended for use in clinical laboratories by Levey and Jennings to judge whether the observed control measurements (or observations) represent reliable or unreliable performance of the analytical method, which in turn may suggest certain sources of error, and so aid in problem solving [9, 10]. Henry, and especially Westgard, ultimately formulated a system of rules to enable clinical laboratory scientists to decide whether the tests they were doing were within acceptable limits and therefore reportable, or outside of acceptable limits [11, 12]. Levey-Jennings charts have been used in laboratories for over 50 years to systematically monitor for errors [9–13]. However, they have not been used in prenatal molecular diagnostic laboratories to establish standardized Levey-Jennings chart-based IQCs for monitoring the quality of molecular diagnostic methods such as FISH in real-time.

We used FISH as a representative method to study the feasibility of establishing Levey-Jennings chart-based IQCs for monitoring molecular diagnostic quality, because FISH is widely used to detect aneuploidies after amniocentesis and to perform chromosome analysis of leukemic blood samples and other tissues [20–24]. In general, each clinical sample only includes one karyotype. However, chimeric samples can contain various karyotypes, including XX/XY sex mosaicism and autosomal trisomy or sex mosaicism and trisomy 13 [25]. Compared with non-chimeric karyotypes, chimeric karyotypes are more common and are more easily misdiagnosed in prenatal diagnosis by FISH, particularly in individuals with a low proportion of multiple karyotypes [24–26]. Thus, considering that the accuracy of aneuploidy detection by FISH can be improved by increasing cell counts [20–22, 27], and that quality-control cells containing different proportions of karyotypes can more accurately assess detection quality [26], we used 60 and 20% as the thresholds for aneuploidy detection [28, 29]. We prepared high-, medium-, and low-value quality-control cells (A-H) with aneuploidy ratios of 10, 30, 70, and 90%, respectively, to provide a relatively consistent ratio of chimeras to the common clinical samples with various karyotypes as the basis to establish an ideal IQC for prenatal molecular diagnosis by FISH [20–24]. Although the quality-control cells transfected with SV40LT are not identical to primary amniocytes and Phaseolus vulgaris-leucoagglutinin (PHA)-stimulated lymphocytes used for FISH quality control approaches in 26 laboratories [30], they have several advantages as IQCs. First, the quality-control cells were made from homologous primary amniotic fluid cells that are used for prenatal testing for aneuploidies. Second, the quality-control cell fluorescence signals were consistent with those of non-cultured primary amniocytes detected by FISH (Fig. 1C). Third, the quality-control cells contain four karyotypes in different proportions with properties similar to chimeric clinical samples, which makes them applicable to evaluating the sensitivity of FISH and convenient for use in quality control. Fourth, quality-control cells prepared from immortalized amniocytes can be effectively produced on a large scale to generate highly biomimetic quality control materials for NIPT [8]. In our study, the results from the detection of trisomy 13 and 21 for quality-control cell (A) were transformed into data that can be used to plot Levey-Jennings charts to establish Levey-Jennings chart-based IQC for aneuploid detection and then monitor whether results are reliable or not [9–13].

As shown in Fig. 2, the Levey-Jennings chart-based IQC for aneuploid detection contains quality control data plotted in the form of a graph in which time (date) is represented on the *x*-axis and the numerical value of the quality control test is represented on the *y*-axis. Moreover, it contains several reference lines, including one for the mean and three on either side of the mean representing standard deviation limits (1 SD, 2 SD, and 3 SD) [9–12]. As shown in Fig. 2c, the Levey-Jennings chart-based IQC demonstrated that the values for six trisomy 13 tests increased gradually over time, and the values for the tests performed on days 5 and 6 exceeded $\overline{\mathrm{X}\ }$ ± 2 SD (outside of acceptable limits) [10–12], which is likely to represent a systematic error caused by control materials, instrument calibration, reagent blanks, or similar factors that affected all measurements in the same way. As shown in Fig. 2d, the day 9 test for trisomy 21 exceeded $\overline{\mathrm{X}\ }$ ± 3 SD on the Levey-Jennings chart-based IQC (outside of acceptable limits) [9–12], which was likely to be a random error introduced by a technician when the sample was being processed. This real-time quality monitoring showed that the test results for the clinical samples on the day that the IQC was performed (outside of acceptable limits) were unreliable. The reasons for this should be identified, and the samples re-tested [10–14].

## Conclusion

The Levey-Jennings chart-based IQC for aneuploid detection can be used to monitor in real-time whether the quality control results are outside of acceptable limits and then determine whether the test results for the clinical samples are reliable, which should be widely applicable for IQC in prenatal molecular diagnostical laboratory.

## Data Availability

All data generated or analyzed during this study are included in this published article.

## Abbreviations

IQC: Internal quality control
FISH: Fluorescence in situ hybridization
SV40LT: Simian virus 40 large T
G418: Geneticin 418
